# 

**Published:** 1893-02-25

**Authors:** 


**Notices of Births, Deaths, and Marriages**, are inserted in this column at a charge of 2s. 6d. for an announcement not exceeding four lines.

### NOTICE TO CORRESPONDENTS.

All MS., Letters, Books for Review, and other matters intended for the Editor should be addressed The Editor, The Lodge, Porchester Square, London, W.

The Editor cannot undertake to return rejected M.S., even when accompanied by stamped directed envelope.

**Notice to Advertisers and Subscribers.**

All Advertisements, Orders for Copies of The Hospital, and Business Communications should be addressed to The Publisher, *not to the Editor*, The Hospital, 140, Strand, W.C.

The Cost of Subscription, post free, is as follows:—Per week, 2½d.; per quarter, 2s. 6d.; per half-year, 5s.; per annum, 8s. 6d. Foreign:—Per annum, 10s. 8d.; payable, in all cases, in advance.

**"Burdett's Hospital Annual," 1893.**

Fourth year of publication. 700 pp. Boards, gilt lettering. A most complete guide to British, Colonial, Indian, and American Hospitals, Dispensaries, Nursing and Convalescent Institutions. and Asylums. Price 5s. Published by The Scientific Press (Limited), 140, Strand, W.C.

**NOTICE.—The Index for Vol. XII. (ending September 1892) is on sale at the Office of this Journal, price 2½d., post free.**

### BOOKS RECEIVED.

SWAN SONNENCHEIN.

"Leprosy and Vaccination." By William Tebb.

"Public Health, and its Applications in Different European Countries." By Albert Palmberg, M.D. Translated.

"How Nature Cures." By Emmet Densmore, M.D. 1892.

W. REEVES.

"Vivisection." By Edward Carpenter and Edward Maitland.

CASSELL AND CO.

"The Year Book of Treatment for 1893."

"Hygiene and Public Health." By B. A. Whitelegge, M.D. Second Edition.

"Elementary Physiology for Students." By A. T. Schofield, M.D.

"In Quest of Gold, or under Whanga Falls." By Alfred St. Johnston.

"Little Folks Christmas Volume."

"The Quiver."

W. H. AND L. COLLINGRIDGE.

"The City Diary for 1893."

SKEFFINGTON and SON.

"Soap-Bubble Stories." By Fanny Barry.

"In the World's Garden." By Maggy Symington.

"Olgar's Dream. A Nineteenth Century Fairy Tale."

"Essays on Vegetarianism." By A. F. Hills.

WHITTAKER AND CO.

"The School Calendar for 1893."

KELLY AND CO.

"Kelly's London Medical Directory."

H. K. LEWIS AND SON.

"A Dissertation on Osteo-Arthritis." By W. H. Russell Forsbrook, M.D., 1893.

"Various Forms of Hysterical or Functional Paralysis." By H. Charlton Bastian, M.D. 1893.

THE SCIENTIFIC PRESS.

"Mental Nursing: A Text-book for Asylum Attendants and Nurses." By William Harding, M.B.

### SCRAPS AND GLEANINGS.

MISS MARGARET GOMBORT is the first woman who received the degree of Doctor of Philosophy and Letters at Brussels.

THE Legislative Assembly of Jersey, after a heated discussion, lasting several hours, refused to allow the optional use of English in that assembly.

IT has been finally settled to reject the motion to permit clinical instruction at the Belfast Union Infirmary, as being contrary to the public feeling on that question.

AN excellent concert was given recently in the children's ward of the Hospital for Diseases of the Throat, Golden Square. The ward was decorated profusely with flowers.

AN extension of the operating buildings of the Brighton Throat and Ear Hospital, is contemplated by the committee, and a fund has been specially started for that purpose.

THE public traffic London horse is not estimated as very long lived; the ordinary omnibus horse rarely lasts more than five years, the tram horse four, and the Post Office horse six.

A REDUCTION of 10 per cent. in miners' wages was agreed to lately by Scotch coal owners. This percentage in the earnings of the colliers is at the rate of a daily loss of 6d. to the men.

THE Tewkesbury Rural Hospital has, for some time, afforded facilities for the access of those medical men who had previously had charge of patients previous to their entering the hospital.

OWING to depression of trade, 42 firms which contributed in 1892 to the Halifax Infirmary and Dispensary, have failed to do so this last year, thus making a decrease in the funds of £184.

LORD ROTHSCHILD has consented to preside at the festival dinner of the Royal Hospital for Diseases of the Chest, City Road, which is to take place at the Hotel Metropole on the 20th June.

AT a meeting of the Worcester Infirmary Committee it was decided to sell out a sufficient amount of the funded property to produce £4,450 in order to complete the purchase of adjoining property.

THE annual report of the Ulster Hospital for Women and Children is a very satisfactory one. The question of a wage limit for free treatment has been under consideration, and is now being tried.

THE Fever Hospital at Kidderminster, which was recently destroyed by fire, was built entirely of wood. The inmates, numbering 28 scarlet fever patients, were rescued with scarcely a minute to spare.

THE Bradford Infirmary Samaritan Society has been proffered the allotment of £100 from the trustees of the Bradford Savings Bank. The number of patients assisted during the year has been 263.

THE annual report of the Suffolk General Hospital reports a large increase in the number of patients, a heavy outlay for repairs, and an unusual number of cases requiring isolation and special nurses:

THE Dean of Norwich has signified his readiness and that of his Chapter, to place the Cathedral at the service of the committee which is engaged in raising funds for the Norfolk and Norwich Hospital.

A FURTHER cheque for £100 has been paid towards the cot in Victoria Hospital for Children, endowed "In memoriam" of the late Duke of Clarence by the efforts of the little members of the Children's Salon.

THE Directors of the Philadelphia Sabbath Association have resolved to issue a call for a State Convention to be held early in February relative to the Sunday closing of the World's Fair and the State Sunday laws.

THE work of the Life-saving Service of America was employed in 337 disasters during last year. The lives of 2,550 persons were saved, and property to the value of £7,111,005 dollars was preserved by the Service.

THE Committee of the Yarmouth Children's Convalescent Home are to be congratulated that only one case of infectious disease has occurred in its midst out of the 211 cases benefited by the institution during the past year.

AT the annual meeting of the Children's Hospital at Bradford, the committee stated that the condition of the establishment was highly satisfactory, and a better report had not been presented since the foundation.

A NUMBER of people were treated in the Royal Hospital, Belfast, for wounds caused in the free fight between the Loyalist and Nationalist parties after the publishing of Mr. Gladstone's speech in Belfast on Monday night.

IT was decided in the French Chamber of Deputies that all amateur cyclists are to pay 10 francs per annum for each tricycle or bicycle that they may own for recreation. This law will come into operation after March 1st next.

THE completion of the Chorley Hospital in connection with the Dispensary is near at hand. Certain specified contributions, which have been received during the year, have been placed as the endowment fund of the hospital.

AT the annual meeting of the Glasgow Royal Infirmary, it was stated that the cases treated in the hospital were 4,000 in excess of those treated in 1891. Only 3,000 citizens contributed £1 yearly, and it was unanimously agreed that the funds of the infirmary must be increased.

A VERY happy evening was spent lately at the Children's Hospital, Hackney Road, when four little lady patronesses, all under twelve years of age, arranged a magic lantern entertainment for the inmates of the Barclay and Duchess of Connaught Wards of that establishment.

A FIRE broke out last week at the Northern Hospital, Great Howard Street, Liverpool, which caused much consternation amongst the patients and attendants. The fire was, fortunately, quickly extinguished without damage to the main buildings. Two nurses acted with much self-possession and heroism on the occasion.

THE finances of the Aberdeen Royal Infirmary are not in a very flourishing condition. More than once the question of closing the hospital has forced itself upon the directors. We cannot imagine for a moment that an adverse balance of an average of £113 on the annual accounts will necessitate such severe measures.

**For Student of Medicine and Medical Appointments see pages VIII. and IX. overleaf.**

### The Student of Medicine.

[All communications for this Supplement should be addressed to The Editor of The Hospital, at 140, Strand, with the words, "Students' Column," written in the left hand top corner of the envelope.]

### PASS LIST.

### Royal Colleges of Physicians and Surgeons of Edinburgh, and Faculty of Physicians and Surgeons of Glasgow.

Final Examination.—Of 108 candidates the following 54 passed, and were admitted L.R.C.P. and S.E., and L.F.P. and S.G.: G. Gibson, Northwich; R. Fairweather, Balfron; J. C. Potter, London; A. F. M. Berkeley, West Indies; R. McConll, Ovington-on-Tyne; H. H. Isherwood, Preston; C. B. Hillyar, Southsea; P. O'Gorman, County Wexford; M. B. Ray, Yorkshire; L. D. Leslie, Margate; A. G. Rolland, North Shields; J. H. Ryan County Limerick; N. J. Billymoria, Bombay; W. H. Anderson, Burton-on-Trent; E. W. Allsom, Liverpool; G. J. Amy, Jersey; A. Phillips, Coventry; W. L. Lovett, Tullamore; E. A. Rowan, Kilkenny; M. Ryan, Cork; G. H. Johnston, Bel[illegible]er; J. E. Binmore Montreal; A. F. Langley Victoria, British Columbia; E. B. Purdon, Belfast; R. Morris, county Cork; H. Campbell, county Antrim; M. P. Coghlan, county Kilkenny; H. A. Jones, county Cork; J. R. Arthur, Canada; J. A. Hislop, Birkenhead; H. Knox, Hillsborough; G. N. Coombes, Madras; W. Wa[illegible]dman, Bingley, Yorkshire; W. T. Hedley, Northumberland; H. C. Swailes, Yorkshire; A. McK. Cleghorn, London, Canada; D. B. Ross, Glasgow; R. Murdoch, Old Cumnock; W. Millar, Leith; J. G. Thomson, Glasgow; G. M. Drury, Cork; R. B. Martin, Carluke; L. Paterson, Harbour Grace, Nfld; W. C. Croxford, Brentford; C. R. Huxley, Kent; T. H. Lawrie, Edinburgh; C. Gilmore, county Down; R. A. Dove, Carlisle; R. D. Jameson, Trinidad; J. Macgown, Cumbrae; M. J. Hickie, county Cork; T. Lawson, Perthshire; G. E. Macleod, India; and A. A. D. Parker, Monmouthshire. Of 43 candidates who entered for the respective divisions 19 passed.

### University of London.

PRELIMINARY SCIENTIFIC (M.B.) EXAMINATION:—Jan., 1893.

Entire Examination.

First Division.—T. W. Edmondson, B.A., Pembroke College, Cambridge and private study; T. H. Gardner, University Tutorial College; S. C. Goodman, B.A., private study and Birkbeck Institution, T. Knowles, B.A., private study and Yorkshire College; E. H. Stevens, B.A., Private study and University Tutorial College, H. F. A. Wigley, B.A., University Tutorial College and private study.

Second Division.—S. Andrade, B.A., Private study; C. T. Bishop, Brighton Grammar School and Charing Cross; N. H. Bonnerjee, Bedford College London and private tuition; A. H. Boyden, B.A., Owens College and Private study; H. N. Coltart, Epsom College and St. George's Hospital; S. J. Coysh, B.A., University Tutorial College; C. V. Crook, B.A., private study; G. C. Owsley, Guy's Hospital; H. Peet, Kingswood School; B. A. Percival, Southampton Boys' College and High School; P. D. Pywell, Private study; Kathleen O. Vaughan, University College Bangor and Bedford College; F. W. Westaway, B.A., private study; H. E. White, Mason College; E. Witham, B.A., Firth College and private study; S. Worthington, B.A., private study.

Chemistry and Experimental Physics.—†B. Amsden, LL.B., B.A., private study; †F. J. Ayre, St Mary's Hospital; †J. S. Barnes, St. Thomas's Hospital and Birkbeck Institution; †H. C. P. Bennett, St. Bartholomew's Hospital; †A. H. Carter, Guy's Hospital; †E. Chatterton, private study; †A. J. Cleveland, Guy's Hospital; †E. H. Coleman, St. John's College, Cambridge; †W. J. E. Davies, private study; †W. N. East, Guy's Hospital; †A. L. Edwards, St. Mary's Hospital; †Mabel Foley, University Tutorial College; †J. A. Glover, Guy's Hospital; †W. A. Golby, St. John's College, Cambridge; A. D. Goodliffe, private study; †H. A. Gunther, University College and private tuition; †Lucy Elizabeth Harris, University College and University Tutorial College; †F. J. Hasslacher, King's College; †H. E. Hewitt, St. Thomas's Hospital; †C. R. Hodgson, Guy's Hospital and private study; F. A. Johns, Hartley Institution and private study; A. Jones, University College, Cardiff; †G. W. G. Jones, St. Mary's Hospital; †J. L. Jones, University College, Aberystwith; †W. M. Jones; private study; †Elizabeth Knight, private study; †Beatrice Knowles, Newnham, Cambridge, and University Tutorial College; R. C. Leaning, Kent College, Canterbury, and private study; †Marion S. Linton, B.A., University College, Bristol, and private study; C. Marriott, Guy's Hospital; †R. Maxwell, private study; †R. W. Mayston, Guy's Hospital and private study; H. L. Norris, St. Thomas's Hospital; †A. W. Oxley, University College; †A. W. Penrose, St. Bartholomew's Hospital and private study; A. G. G. Plumley, Clifton Laboratory; †J. H. Rhodes, Hilderthorpe School, Scarborough, and private tuition; †A. M. Ross, King's College; †H. A. Scholberg, St. Bartholomew's Hospital, H. D. Singer, Merchant Taylors' School; †A. B. Smallman, Owens College, †J. E. Smith, University College, Liverpool; †F. E. Taylor, Yorkshire College; †J. W. Turner, private study; †Elinor Adeline N. Twigg, Mason College and private study; †W. F. Tyndale, St. George's Hospital and Birkbeck Institution; †W. P. Walker, Owens College; †G. B. Webb, Guy's Hospital; †Charlotte L. Weetman, University Tutorial College; †H. C.

### MEDICAL APPOINTMENTS.

CITY OF LONDON HOSPITAL FOR DISEASES OF THE CHEST, Victoria Park, E.

Applications, with testimonials, for the POST of HOUSE PHYSICIAN are invited to be sent to the Secretary, at the Office, 24, Finsbury Circus, E.C., not later than March 9th, 1893.

Board and residence, and allowance for washing provided.

91, West Regent Street, Glasgow. (609)

### THE STUDENT OF MEDICINE—(*continued*).

† These Candidates have now completed the Examination.

### VACANCIES.

**The following vacancies are announced this week, the amount of salary in each case being given after the name of the institution:**

#### Resident Medical Officers.

**Buckinghamshire General Infirmary, Aylesbury. Resident Surgeon and Apothecary. Unmarried. Doubly qualified. Salary £80 for the first year, rising £10 annually to £100, with board, washing, coals, and candles, unfurnished apartments. Applications to Mr. George Fell, Solicitor, Aylesbury, by February 27th.**

**City of London Hospital for Diseases of the Chest, Victoria Park, E. House Physician. Board, residence, and allowance for washing provided. Applications to the Secretary, at the office, 24, Finsbury Circus, E.C., by March 9th.**

County Asylum, Prestwich, Manchester. Junior Assistant Medical Officer, unmarried. Salary £100 per annum, with prospect of increase of £25 at the end of the first year, and a similar increase at the end of the second year, with further increase according to promotion, with furnished apartments, board, washing, and attendance. Applications to the Superintendent.

London Temperance Hospital, Hampstead Road, N.W. House Surgeon. Doubly Qualified. Appointment for six months. Salary at the rate of 50 guineas per annum, with residence in the hospital, board and washing. Applications to E. Wilson Taylor, Secretary, by March 3rd.

London Temperance Hospital, Hampstead Road, N.W. Junior House Surgeon. Appointment for six months. Residence in the hospital board and washing provided, and an honorarium of 5 guineas at satisfactory completion of term of appointment. Applications to E. Wilson Taylor, Secretary, by March 3rd.

Nottingham General Dispensary, Broad Street, Nottingham. Assistant Resident Surgeon, doubly qualified. Salary £150 per annum with rooms and attendance. Applications to Charles H. Preston, Secretary, by March 6th.

Salford Royal Hospital, Salford. House Surgeon. Salary £100 per annum, with board, and residence. Applications to the Secretary, by February 25th.

York Dispensary. Resident Medical Officer, unmarried. Salary £150 per annum, with furnished apartments, coals, and gas. Applications to S. W. North, Esq., 84, Mickelgate, York, by February 28th.

#### Non-Resident Medical Officers.

Guy's Hospital, S.E. Assistant Dental Surgeon. Applications to the Dean, by March 4th.

Royal South London Dispensary, St. George's Cross, S.E. Honorary Surgeon. Applications to the Committee of Management by February 28th.

St. John's Hospital for Diseases of the Skin, Leicester Square, W.C. Assistant Physician. Applications to St. Vincent Mercier, Secretary.

St. John's Wood and Portland Town Provident Dispensary, 1, Hensbridge Villas, St. John's Wood, N.W. Third Medical Officer, doubly qualified. Applications to the Secretary by February 27th.

St. Mark's Hospital for Fistula and other Diseases of the Rectum, City Road, E.C. Anæsthetist. Honorarium £50 per annum. Applications to the Secretary by February 25th.

University of Glasgow. Four examinerships for degrees in Medicine. Annual fee, £30. Applications to Alan E. Clapperton, Secretary, to the Court 91, West Regent Street, Glasgow, by March 11th.

